# A145 PEDIATRIC PATIENTS’ & PARENTS’ PERSPECTIVES ON TREATMENT PREFERENCE IN EOSINOPHILIC ESOPHAGITIS: A CROSS SECTIONAL, QUALITATIVE RESEARCH STUDY

**DOI:** 10.1093/jcag/gwac036.145

**Published:** 2023-03-07

**Authors:** A Sethuraman, E Chan, J Jia, L Soller, S Erdle, V Avinashi

**Affiliations:** Division of Allergy and Immunology, BC Children's Hospital, Vancouver, Canada

## Abstract

**Background:**

Eosinophilic esophagitis (EoE) is a chronic inflammatory disease of the esophagus which impairs quality of life in children and adolescents. Given the wide variety of symptomatic presentations in pediatrics, the varying time to diagnosis, and differing severity (including stricturing phenotype) initial treatments are not standardized. An important part of this individualization, beyond the health care practitioner counseling involves the patients’ and parents’ preferences which incorporates personal beliefs and attitudes.

**Purpose:**

The study aim was to qualitatively describe parental and patient preferences regarding treatment options in EoE.

**Method:**

This was a cross-sectional qualitative research study conducted in the EoE clinic at BC Children’s Hospital, Vancouver. Parents and their children completed semi-structured survey questions regarding treatment preference in EoE. Patients along with their parents who completed their survey questions were included in the study. Incomplete forms were excluded from the study.

**Result(s):**

The survey was attempted by 15 children, 7-11yrs of age, and 42 children, 12-18yrs of age, along with their parents. Completed response by both parent and their children were seen in 47 patients, 40 were adolescents 12-18yrs of age and 7 were 7-11yrs of age. Parent treatment preferences were diet in 17/47(36.2%), medications in 21/47 (44.7%) and 9/47 (19%) were unsure. 75% of parents who preferred dietary management thought it was less risky than medication. Parents who chose medication thought it would be easier (8/21) and more effective (8/21). Most of the parents’ decisions were influenced by the physician (35/47; 74.5%) and more so by the gastroenterologist (28/47; 59.6%). In 12–18-yr olds, 6/40 (15%) had preference for diet, 25/40 (62.5%) preferred medication and 9/40 (22.5%) were unsure. 3/6 adolescents thought that diet was less risky and 4/6 thought it was more effective than medication. Among the teens who preferred medication, 17/25 (68%) felt it to be easier and 9/25 (36%) thought it would work better than diet. Teenagers found their parents to be helpful for deciding (50%) and doctor (50%) with the gastroenterologist again playing a prominent role. In the 7 to 11yr old age group, 4/7 (57.1%) predominantly had preference for dietary treatment.

**Conclusion(s):**

The overall preference in the adolescent age group is for medication supported by patients’ and parents’, with ease of use being a primary driver for adolescents, whereas ease of use and effectiveness were drivers for the parents. Parents were more often interested in dietary therapy than the adolescents. With regards to parents of younger children and younger children themselves, dietary management was the preference as they felt it to be less risky. The numbers, though small, represent one of the few studies done on patient preference in EoE. Future studies should include formal qualitative studies and preferences could ultimately be tied to better counseling and tracking adherence to therapy.

**Please acknowledge all funding agencies by checking the applicable boxes below:**

None

**Disclosure of Interest:**

None Declared

